# To touch or to be touched? comparing appraisal of vicarious execution and reception of interpersonal touch

**DOI:** 10.1371/journal.pone.0293164

**Published:** 2024-05-17

**Authors:** Niccolò Butti, Cosimo Urgesi, Francis P. McGlone, Viola Oldrati, Rosario Montirosso, Valentina Cazzato

**Affiliations:** 1 Scientific Institute, IRCCS E. Medea, 0–3 Centre for the at-Risk Infant, Bosisio Parini, Lecco, Italy; 2 Department of Life Sciences, PhD Program in Neural and Cognitive Sciences, University of Trieste, Trieste, Italy; 3 Scientific Institute, IRCCS E. Medea, Lecco, Italy; 4 Laboratory of Cognitive Neuroscience, University of Udine, Udine, Italy; 5 Department of Neuroscience and Biomedical Engineering, Aalto University School of Science, Espoo, Finland; 6 Faculty of Health, School of Psychology, Liverpool John Moores University, Liverpool, United Kingdom; 7 Department of Cognitive, Psychological and Pedagogical Sciences and Cultural Studies, University of Messina, Messina, Italy; Anglia Ruskin University, UNITED KINGDOM

## Abstract

Unmyelinated C-Tactile (CT) fibres are activated by caress-like touch, eliciting a pleasant feeling that decreases for static and faster stroking. Previous studies documented this effect also for vicarious touch, hypothesising simulation mechanisms driving the perception and appreciation of observed interpersonal touch. Notably, less is known about appreciation of vicarious execution of touch, that is as referred to the one giving gentle touch. To address this issue, 53 healthy participants were asked to view and rate a series of videoclips displaying an individual being touched by another on hairy (i.e., hand dorsum) or glabrous (i.e., palm) skin sites, with touch being delivered at CT-optimal (5 cm/s) or non-CT optimal velocities (0 cm/s or 30 cm/s). Following the observation of each clip, participants were asked to rate self-referred desirability and model-referred pleasantness of vicarious touch for both executer (toucher-referred) and receiver (touchee-referred). Consistent with the CT fibres properties, for both self-referred desirability and model-referred pleasantness judgements of vicarious touch execution and reception, participants provided higher ratings for vicarious touch delivered at CT-optimal than other velocities, and when observed CT-optimal touch was delivered to the hand-dorsum compared to the palm. However, higher ratings were attributed to vicarious reception compared to execution of CT-optimal touch. Notably, individual differences in interoceptive trusting and attitude to interpersonal touch were positively correlated with, respectively, toucher- and touchee-related overall appraisal ratings of touch. These findings suggest that the appreciation of both toucher- and touchee-referred vicarious touch is specifically attuned to CT-optimal touch, even though they might rely on different neurocognitive mechanisms to understand affective information conveyed by interpersonal tactile interactions.

## Introduction

Interpersonal tactile interactions are pervasive in the early stages of life and play a pivotal role in forming social bonds and communicating emotional information throughout the life span [1,2]. At a somatosensory level, touch pleasantness correlates with the activity of slow-unmyelinated afferents, called C-Tactile (CT) fibres [3,4]. CT fibres, predominantly innervating hairy skin, respond optimally to touch delivered at velocities of 1–10 cm/s, at a temperature similar to human skin [5,6], eliciting a pleasant sensation that decreases for faster and slower touch [7,8] (but see Croy et al., 2021 for evidence on large individual variability of subjective pleasantness). Through their projections to the dorsal posterior insula [9,10], CT afferents would support the processing of affective information entailed by social touch. Accordingly, recent research has documented the functional role of the CT afferents in affect regulation and social development [11], highlighting, for instance, the effects of affective touch reception in modulating parasympathetic activation [12,13], in reducing pain [14–16], and in buffering feelings of social exclusion [17].

Beyond the somatosensory effects of touch, there is evidence of affective responses also when interpersonal gentle touch is just ‘observed’. Within the framework of the ‘Embodied simulation theory’ of touch [18], individuals can map others’ tactile events by re-using their own motor, somatosensory and visceromotor representations. As a result, this tactile mapping would allow an observer to perceive the touch as if that person was receiving the same kind of tactile stimulation, thus facilitating the understanding of how the other person is ‘feeling’ that touch [19,20]. In line with this, functional neuroimaging evidence reports the activation of the dorsal posterior insula during vicarious observation of CT-optimal touch [21], pointing to a similar hedonic response to directly felt and vicarious touch experiences. Furthermore, observation of vicarious interpersonal touch is rated as more pleasant when delivered at CT-optimal compared to non-CT optimal velocities (i.e., slower or faster touch) [20,22–25]. These findings suggest that the human brain may be attuned to “see” CT-specific features when watching others performing interpersonal touch actions [21].

Little is known, however, about the vicarious representation of *giving* gentle touch. Indeed, observing others’ actions triggers a simulative representation of the observed action, which includes not only movement kinematics, but also the affective valence of movement [26–29]. At a higher-order socio-affective level, affective touch may have important social meaning for both those who give and those who receive it [30]. Evidence suggests that affective touch has a beneficial effect also for the person delivering the stroking gesture (i.e., toucher), given that those who give touch may convey feelings of closeness and care toward a touchee, who in turn may feel bonded and safe [31,32]. In primates, social interactions involving social touch are of critical importance for group life, so that delivering social touch is associated with desirable individual and social benefits [33]. Furthermore, stroking other’s skin is perceived as pleasant by the toucher [34], is associated with more positive sensory experiences when delivered at CT-optimal velocities [35], and is spontaneously targeted to activate CT afferents [36].

Despite these similarities between touch receiving and giving, there are also profound perceptual differences which arise from the body parts that an individual normally uses to give and receive touch. Typically, most of the touching actions, like hand-holding, cradling and embracing, are performed with the toucher’s palm contacting a touchee’s arm, shoulder, or back [34,37]. Notably, while CT afferents are widely represented in the hairy skin, the palm is densely innervated by fast-conducting Aβ fibres rather than CT afferents [38]. Thus, the tactile systems might receive more readily benefits from CT signalling when activated by touch receiving than by touch execution [30], suggesting a preference for reception over the hairy skin, compared to execution with the palm, of affective touch [34]. Accordingly, previous research documented that vicarious preferences to observed interpersonal touch match not only the optimal velocities, but also the anatomical distribution of CT afferents [23].

Taken together, all these studies corroborate the importance of interpersonal gentle touch for positive tactile interactions and suggest that the experiences and consequences of affective touch may differ between those who give and those who receive touch, even when touch is only observed. Yet, the commonalities and differences between the vicarious appreciation of touch giving and of touch receiving are unclear.

The present study sought to fill this theoretical gap by investigating toucher- and touchee-related appraisal ratings of interpersonal CT-optimal touch. Specifically, the focus in this report is on how ‘observed’ interpersonal touch is perceived as referred to the person receiving (i.e., touchee) or giving (i.e., toucher) the touch.

In line with previous studies [23,24], two different questions were used to compare the affective appraisal of touch giving and touch receiving. Specifically, we asked participants to judge how much they would like to touch/to be touched like that and how the observed touch was pleasant for the person touching/being touched. Indeed, the first question tapped into how desirable was for each individual to give or receive the observed touch, while the second question referred to the perceived pleasantness for the model. The rationale for this manipulation is that the appraisal of model-referred pleasantness of touch might rely more on the learned expectations about the rewarding value elicited by interpersonal stroking [20,39]. That is, even though one may not like to be stroked, they should still be able to acknowledge the hedonic, universally recognised, positive value of interpersonal touch. Consistent with this view, previous studies documented higher ratings for the model-referred pleasantness question compared to the question assessing desirability of receiving the observed touch [23,24]. To the best of our knowledge, no studies so far has compared these two appraisal dimensions on vicarious social touch execution.

A second aim of the study was to explore individual differences in interpersonal touch due to childhood experiences and attitudes, and interoceptive awareness, as these might facilitate (or hinder) the experience of vicarious social touch [24,40]. The negative effects of childhood neglect/abuse and later life experiences on perception of affective touch are well known [41,42], with a recent study reporting blunted responses to vicarious affective touch in young adults who have experienced early life adversity and consequently spent time in foster care [22]. Furthermore, awareness of internal bodily states, namely the sense of interoception, may play a role in vicarious social touch, with individuals with higher levels of interoceptive awareness found to show higher responses in somatosensory areas for vicarious touch perception [43].

In line with previous evidence, for both toucher- and touchee-referred ratings, overall higher appraisal was expected for gentle touch delivered to the hairy skin (i.e., hand), compared to the glabrous skin (i.e., palm), and at CT-optimal, compared to non-CT optimal velocities. Importantly, given that touchers and touchees may differ in the comfort they gain from interpersonal touch, higher appreciation of affective touch reception compared to execution should be found. Finally, individual differences in social touch experiences and attitudes, as well as in interoceptive awareness, would be linearly associated with toucher- and touchee-related appraisal ratings.

## Materials and methods

### Participants

Based on a Repeated Measure ANOVA model with two agents, two questions, two body sites, and three stroking velocities, an a-priori power analysis using the G*Power 3.0.10 software [44] indicated that a sample > 50 allowed detecting a large effect size (*n*^*2*^_*p*_ = 0.14), with 95% power and alpha set at 0.05 (two tailed). Hence, 53 participants (31 females, 22 males; age mean = 28.6 years, SD = 4.8) completed all the procedures of the study. Of these, 45 individuals participated remotely, while 8 participants completed the experiment in our laboratory at Liverpool John Moores University (LJMU). For the online participation, a total of 56 individuals preliminarily signalled their availability, but 11 participants did not complete the procedures (drop-out rate = 20%). Although it was not possible to collect detailed demographic information on people who dropped out, age and sex (6 females, 5 males; age mean = 27.5 years, SD = 4.3) were similar to those of the overall sample, limiting the risk of selection bias. The lab-based sample was composed of 4 PhD students and 4 undergraduate students from the LJMU, with a prevalence of male participants and a mean age in the early twenties (3 women, 5 men; age mean = 23 years, SD = 5.5). Participants who completed the study online were recruited from regions of the northern Italy, with a prevalence of female individuals and a mean age in the late twenties (28 women, 17 men; age mean = 29.6 years, SD = 4). All of them had completed secondary education, with 22 individuals having a bachelor’s or master’s degree, 10 having a PhD or being PhD students, and 7 being undergraduate students. Despite differences in demographic features and the expected high individual variability [45], the lab-based and the online-based participants showed comparable ratings across task conditions (S1 and S2 Tables in S1 File), reassuring that data obtained from both modalities could be analysed together. It is worth noting that, for the vicarious touch task, online collected vicarious touch ratings were reported to be comparable to those of lab-based studies [46]. Accordingly, a recent study analysed together vicarious touch ratings obtained from 253 online-based participants and from 31 lab-based participants [47].

Participants were recruited through posters, social media advertisements, and emails to research panel lists (i.e., lists of people who have previously signalled their availability to participate in research studies). Inclusion criteria were: i) having normal or corrected to normal vision (with glasses/contact lenses) ii) being right-handed, iii) having no history of or any form of neurological and psychiatric disorders, iv) having no history of or any clinical condition of chronic pain and skin diseases. Participants for the lab-based study were compensated for their time with a £5 gift voucher and granted course credits if undergraduate psychology students. Online participants did not receive any reimbursement. All procedures were approved by the LJMU Research Ethics Committee (reference number: 22/PSY/078) and complied with the ethical standards of the 1964 Declaration of Helsinki.

Informed consent was obtained by asking all participants to tick the relative box after reading the participant information sheet. Doing so, participants were asked also to confirm that they matched the inclusion criteria. The study recruitment started on the 12^th^ of February and was completed by the 16^th^ of April 2023.

### General procedure

All participants were asked to complete a vicarious affective touch task through the E-Prime 3® software (Psychology Software Tools, Pittsburgh, PA, USA), which allowed controlling stimuli administration and randomisation. The E-Prime Go® package was used for remote data collection. Then, self-report questionnaires were administered via Qualtrics® (Provo, UT, USA). Background information (e.g., age, sex) was also collected, and participants were asked to confirm that they were right-handed by completing the Edinburgh handedness inventory [48]. Only right-handed individuals were recruited as the displayed touchers’ movements in the vicarious social touch task were all executed with the right hand, thus potentially triggering simulative representations for the same hand [49]. Online participants were asked to sit in a quiet room and to complete the task and questionnaires in a single session. All procedures required about 30 minutes to be completed. Upon completion of the procedure participants were debriefed about the aims and instruments of the study.

### Vicarious touch rating task

An adapted version of a previously published task was administered [23,24]. The task consisted of 6-second-long videos of both males and females applying touch with their right hand to female and male actors. Touch was delivered with CT-optimal (5 cm/s) and non-CT optimal velocities (static: 0 cm/s, fast: 30 cm/s) on the hand-dorsum and on the palm. These two body regions were selected as they were matched in terms of size and, thus, observed movements, whereas they represented areas with different density of CT-fibres, respectively, a hairy and a glabrous skin site. Moreover, the hand is considered a body part that strangers are allowed to touch [40,50], thus appraisal ratings of this site should be less influenced by top-down modulations related to the toucher’s identity.

Each trial started with a white fixation cross on a black background lasting 1 second, followed by the 6-second-long video presentation. At the end of each video, one of four questions were presented in the middle of the screen, written in white letters on a black background. These questions concerned touch delivering and reception and were designed to probe expectations of how the observed touch was perceived in terms of self-referred desirability or model-referred pleasantness. Under the question participants were presented with a white Visual Analogue Scale (VAS) scale ranging from 0 = “Not at all” to 100 = “Extremely”, for self-referred desirability, and from 0 = “Very unpleasant” to 100 = “Extremely pleasant”, for model-referred pleasantness. Participants were asked to choose their answer by moving the cursors in the desired position of the VAS. After a response was given, the next trial started.

The questions were as follows: for the toucher, “How much would you like to touch like that?” (self-referred desirability) and “How pleasant do you think that action was for the person touching?” (model-referred pleasantness); for the touchee, “How much would you like to be touched like that?” (self-referred desirability) and “How pleasant do you think that action was for the person being touched?” (model-referred pleasantness). In the Italian version these questions were translated as follows: “Quanto ti piacerebbe toccare in questo modo?” (self-referred desirability for the toucher), “Quanto pensi sia stata piacevole l’azione per chi ha toccato?” (model-referred pleasantness for the toucher), “Quanto ti piacerebbe essere toccato in questo modo?” (self-referred desirability for the touchee), “Quanto pensi sia stata piacevole l’azione per chi è stato toccato?” (model-referred pleasantness for the touchee). The translation respected the conceptual and linguistic differences between the hypothetical, motivational-based, future-oriented sentences for the desirability question, and the actual, hedonic-based, past-oriented sentences for the model-referred pleasantness question. In Italian, the VAS scale ranged from 0 = “Per nulla” to 100 = “Moltissimo”, for self-referred desirability, and from 0 = “Molto spiacevole” to 100 = “Molto piacevole”, for model-referred pleasantness.

Separate blocks were administered for the toucher and the touchee, and within each block, the desirability and pleasantness questions were presented in separate blocks, for a total of 4 blocks. At the beginning of the experiment, participants read an instruction slide informing them that they would be presented with a series of videoclips, and that at the end of each video they would be asked to answer moving the mouse on a VAS from 0 to 100. They were also asked to answer as truthfully as possible and to remember that there was no correct or wrong answer. At the beginning of each block a new instruction slide told participants to watch the videos and to answer to the specific question. The task structure in blocks and the corresponding questions are reported in Table 1.

**Table 1:** Task structure and questions.

		Agent	
		Toucher	Touchee
**Question**	**Desirability**	*“How much would you like to touch like that*?*”*	*"How much would you like to be touched like that*?*”*
	**Pleasantness**	*“How pleasant do you think that action was for the person touching*?*”*	*“How pleasant do you think that action was for the person being touched*?*"*

**Fig 1:**
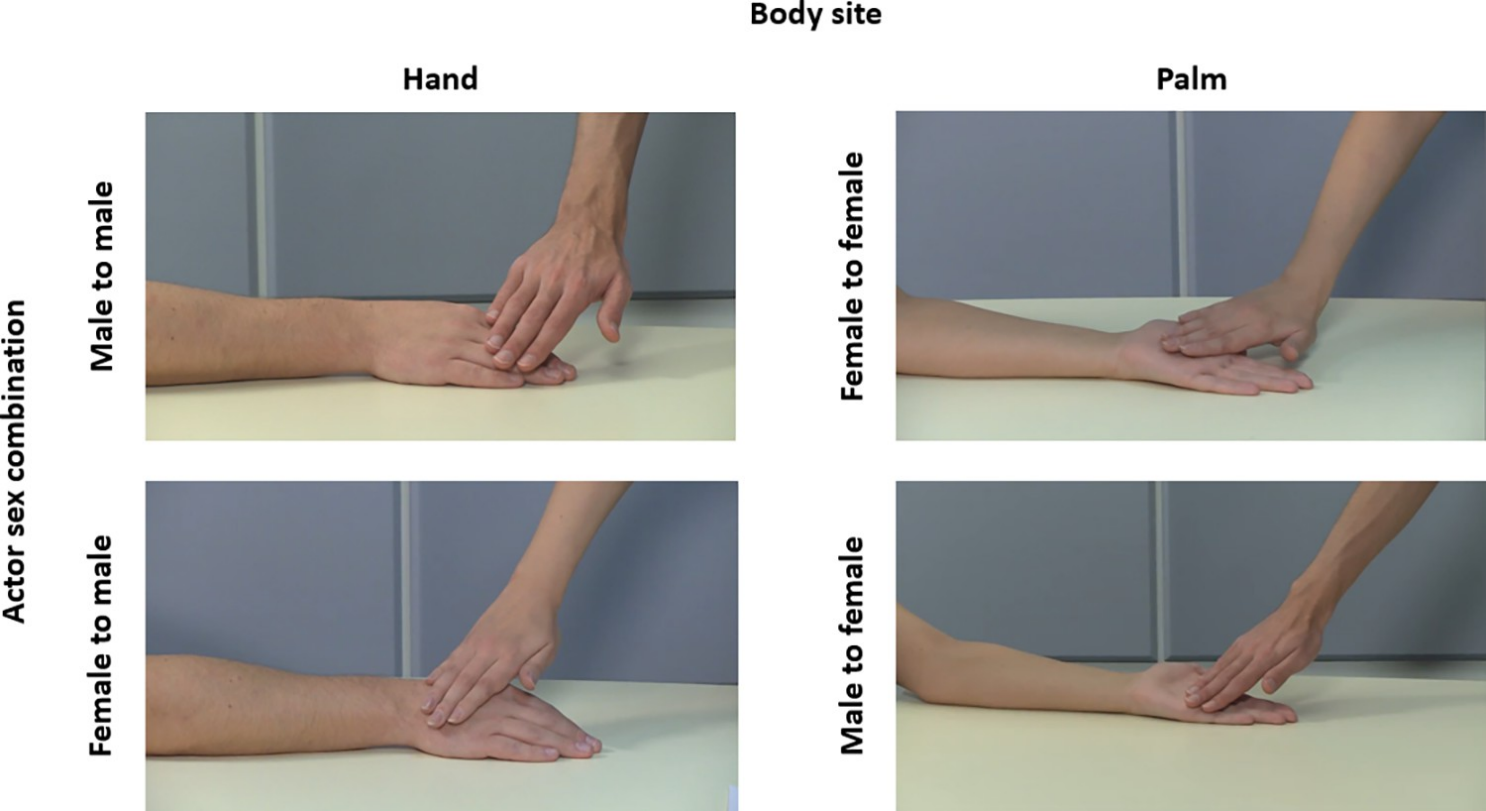
Examples of video stimuli representing touch delivered on two body sites with all combinations of biological sex of actors.

The order of administration of the four blocks was counterbalanced amongst participants. Considering a 2 agent × 2 question × 2 body site × 3 velocity design, 24 videos were presented once within each block in a completely randomised fashion. These videos represented all possible combinations of biological sex of actors with body sites and velocities. Overall, across all conditions and blocks, a total of 96 (i.e., 24 videos x 4 blocks) videos was presented. Examples of video stimuli are reported in Fig 1.

### Self-report questionnaires

#### Multidimensional Assessment of Interoceptive Awareness

The Multidimensional Assessment of Interoceptive Awareness (MAIA) [51] is a 32‐item questionnaire which investigates eight dimensions of interoceptive bodily awareness: noticing (4 items), not distracting (3 items), not worrying (3 items), attention regulation (7 items), emotional awareness (5 items), self-regulation (4 items), body Listening (3 items) and trusting (3 items). Questionnaires are answered using a 6-point Likert scale ranging from 0 = “Never” to 5 = “Always”. Each individual dimension is scored by the average of scores from questions corresponding to that subscale, with some questions being reversed scored. Good internal consistency was reported for the MAIA questionnaire (Cronbach α = 0.90) [52]. For this study, the noticing and trusting scales were selected, as these variables were more congruent with the hypothesis that the more a person is aware of the own bodily signals, the more this person may feel the sensations elicited by observed interpersonal touch [53].

#### Touch experiences and attitudes questionnaire

The short 37-item version of the Touch Experiences and Attitudes Questionnaire (TEAQ) [54,55] assesses current and childhood experiences of positive touch and an individual’s attitude towards interpersonal touch. Questions are answered using a 5-point Likert scale ranging from 1 = “Disagree strongly” to 5 = “Agree strongly”. A mean score is calculated for each of the five subscales: attitude to friend and family touch (7 items), attitude to intimate touch (10 items), childhood touch (8 items), attitude to self-care (7 items), and current intimate touch (5 items), with negatively worded questions reversed scored. The TEAQ short version was found to have a good internal consistency (Cronbach α = 0.93). In line with our hypothesis that individual experiences and attitudes towards interpersonal touch should bias appraisal ratings of vicarious touch, the childhood touch and the attitude to friend and family touch scales were selected as variables of interest, excluding the others as they focused on stroking the one own body and intimate touch, aspects that were less compatible with the adopted videos.

### Data handling and statistical analysis

The appraisal ratings attributed to each condition of the vicarious touch task were inserted into a Repeated Measure (RM) ANOVA with 2 agent (toucher vs. touchee) × 2 question (desirability vs. pleasantness) × 2 body site (hand vs. palm) × 3 velocity (3 levels: 0 cm/s, 5 cm/s and 30 cm/s) as within-subject variables.

In keeping with previous research [24,36], two indexes of touch appraisal were calculated, namely the Overall Touch Appraisal (OTA) and the Pleasant Touch Awareness (PTA). Since agent (i.e., touch vs. touchee) and question (i.e., desirability vs. pleasantness) represented the two main manipulations of the task, the OTA and PTA were calculated separately for each agent and question. Indeed, for each of the four conditions (i.e., toucher, touchee, desirability, pleasantness) the appraisal ratings across the different conditions of the other variable (i.e., agent, question) were collapsed into a single value, regardless of the two body sites (i.e., palm, hand) while considering separately the three different velocities. As an example, for the toucher condition, ratings in the desirability- and pleasantness-question blocks for both the hand and the palm were collapsed, obtaining an averaged value of the appraisal ratings expressed for the toucher separately for static, CT-optimal and fast touch. Then, the OTA was computed as the average rating across the three velocities, thus representing an index of individual appraisal of interpersonal touch that is not CT-specific. As a proxy of individual preference towards CT-optimal velocity, the PTA index was calculated using the following formula: (CT-optimal–non-CT optimal fast velocity)/OTA. Spearman’s *r* correlations were run between both OTA and PTA corresponding to the four different conditions, and the selected scales of the questionnaires, namely, noticing and trusting for the MAIA, and attitude to friend and family touch and childhood touch for the TEAQ.

All analyses were performed with Statistica 8.0 (Statsoft, Tulsa, OK), and data were reported as Mean ± Standard Error of the Mean (SEM). The significance threshold was set at *p* < 0.05 for all effects. Significant interactions were analysed with Duncan’s post-hoc test correction for multiple comparisons, which allows testing effects of different size in the same design [56,57]. For the correlations, the Bonferroni correction was adopted to adjust the standard *p*-value according to the number of comparisons (corrected *p* = 0.013). Effect sizes were estimated and reported as partial eta squared (*n*^*2*^_*p*_) for ANOVA designs, adopting conventional cut-off of 0.01, 0.06, and 0.14 for small, medium, and large effect sizes, respectively, and as Cohen’s d for pairwise comparisons, adopting conventional cut-off of 0.2, 0.5, and 0.8 for small, medium, and large effect sizes, respectively [58].

## Results

**Fig 2:**
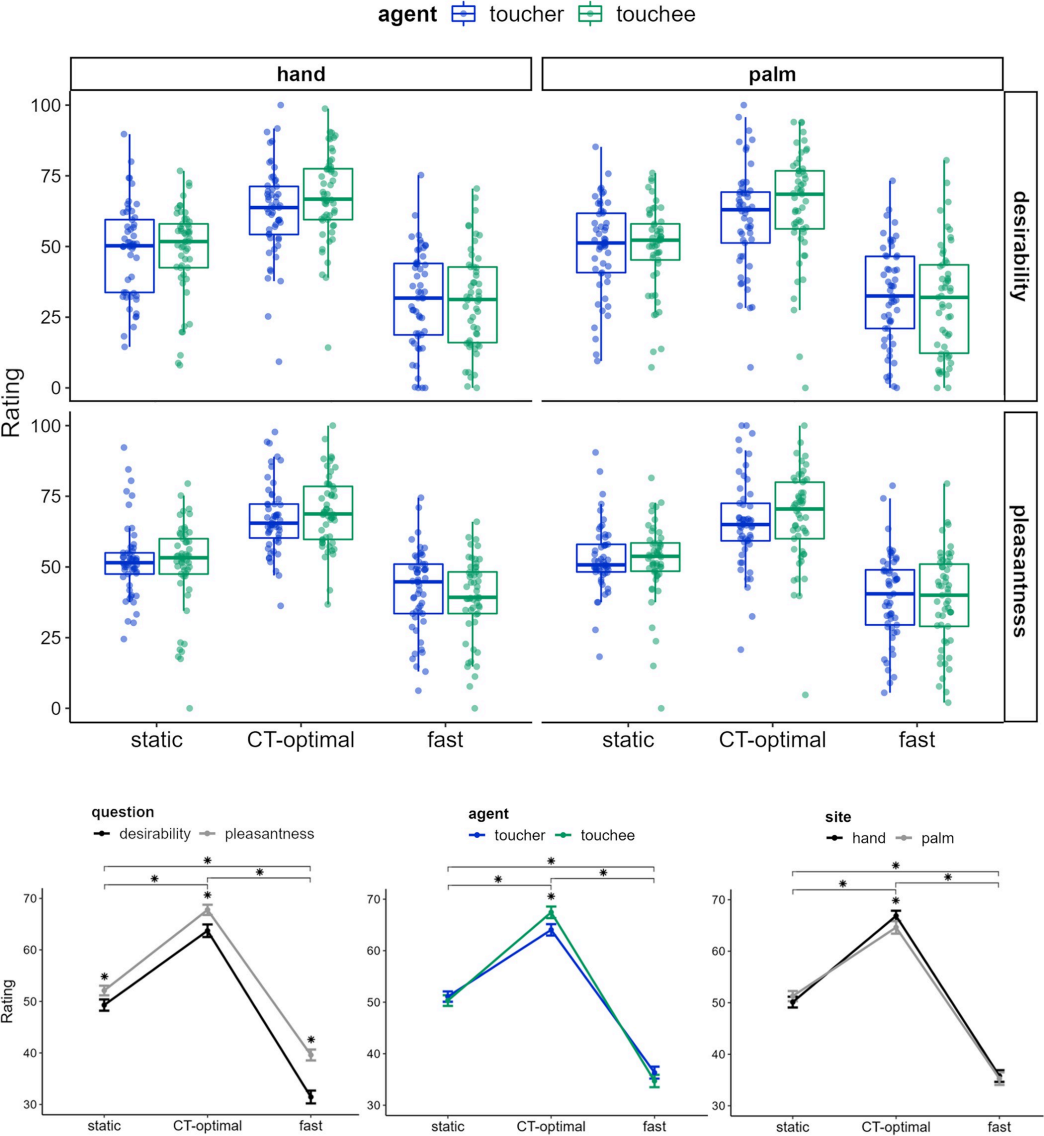
Boxplot of pleasantness ratings in the vicarious touch task and line graphs of the two-way significant interaction effects. Dots represent observations; asterisks indicate the velocity level at which the interaction effects were significant.

The analysis yielded significant main effects of the two-level question factor (desirability: 48.15 ± 1.32, pleasantness: 53.16 ± 0.98; *F*_*1*,*52*_ = 39.51, *p* < 0.001, *n*^*2*^_*p*_ = 0.43) and the three-level velocity variable (0 cm/s: 50.70 ± 1.63, 5 cm/s: 65.75 ± 1.74, 30 cm/s: 35.52 ± 1.86; *F*_*2*,*104*_ = 81.72, *p* < 0.001, *n*^*2*^_*p*_ = 0.61), which were further qualified by a significant 2-way interaction of question × velocity (*F*_*2*,*104*_ = 5.67, *p* = 0.005, *n*^*2*^_*p*_ = 0.10). Post-hoc tests indicated that, across questions, CT-optimal touch was preferred than both non-CT optimal velocities, with fast stroking being judged as less pleasant than static touch (all *p* < 0.001; all Cohen’s d > 1.01). In addition, across velocities, higher ratings were attributed to model-referred pleasantness compared to self-referred desirability (all *p* < 0.018; Cohen’s d: static = 0.23, CT-optimal = 0.30, fast = 0.56). Moreover, a significant agent × velocity interaction (*F*_*2*,*104*_ = 5.23, *p* = 0.007, *n*^*2*^_*p*_ = 0.09) revealed lower appraisal for toucher- than touchee-referred ratings only for CT-optimal velocity (5 cm/s: 64.05 ± 1.88 vs. 67.45 ± 1.82; *p* = 0.005; Cohen’s d = 0.25), while such a difference did not emerge for static (0 cm/s: 51.09 ± 1.74 vs. 50.32 ± 1.80; *p* = 0.513; Cohen’s d = 0.06) and fast touch (30 cm/s: 36.34 ± 1.91 vs. 34.71 ± 2.08; *p* = 0.169; Cohen’s d = 0.11). Preferences for CT-optimal stroking compared to non-CT optimal velocities, and for static compared to fast touch, were detected for either agent (all *p* < 0.001; all Cohen’s d > 0.98). The velocity × body site interaction was also significant (*F*_*2*,*104*_ = 4.24, *p* = 0.017, *n*^*2*^_*p*_ = 0.08), with a preference for touch received on or delivered to the hand compared to the palm detectable only for CT-optimal velocity (5 cm/s: 66.87 ± 1.61 vs. 64.64 ± 2.13; *p* = 0.008; Cohen’s d = 0.16), and no differences between body sites for static (0 cm/s: 50.12 ± 1.78 vs. 51.28 ± 1.65; *p* = 0.162; Cohen’s d = 0.09) and fast touch (30 cm/s: 35.78 ± 1.85 vs. 35.27 ± 2.00; *p* = 0.539; Cohen’s d = 0.04). Neither the 3-way agent × velocity × body site nor any other main and interaction effects were significant (all *F* < 1.87, all *p* < 0.159). The appraisal ratings attributed to each condition and the significant two-way interactions are represented in Fig 2.

To sum up, for both desirability and pleasantness ratings of touch execution and reception, participants expressed higher appraisal for touch delivered at CT-optimal vs. non-CT optimal velocities, and when CT-optimal touch was delivered to the hand-dorsum compared to the palm. Furthermore, higher appraisal was attributed to touchee- compared to toucher- referred ratings only for CT-optimal touch velocities (5 cm/s). Finally, the model-referred pleasantness ratings were higher than the desirability ratings.

### Correlation analyses

**Fig 3:**
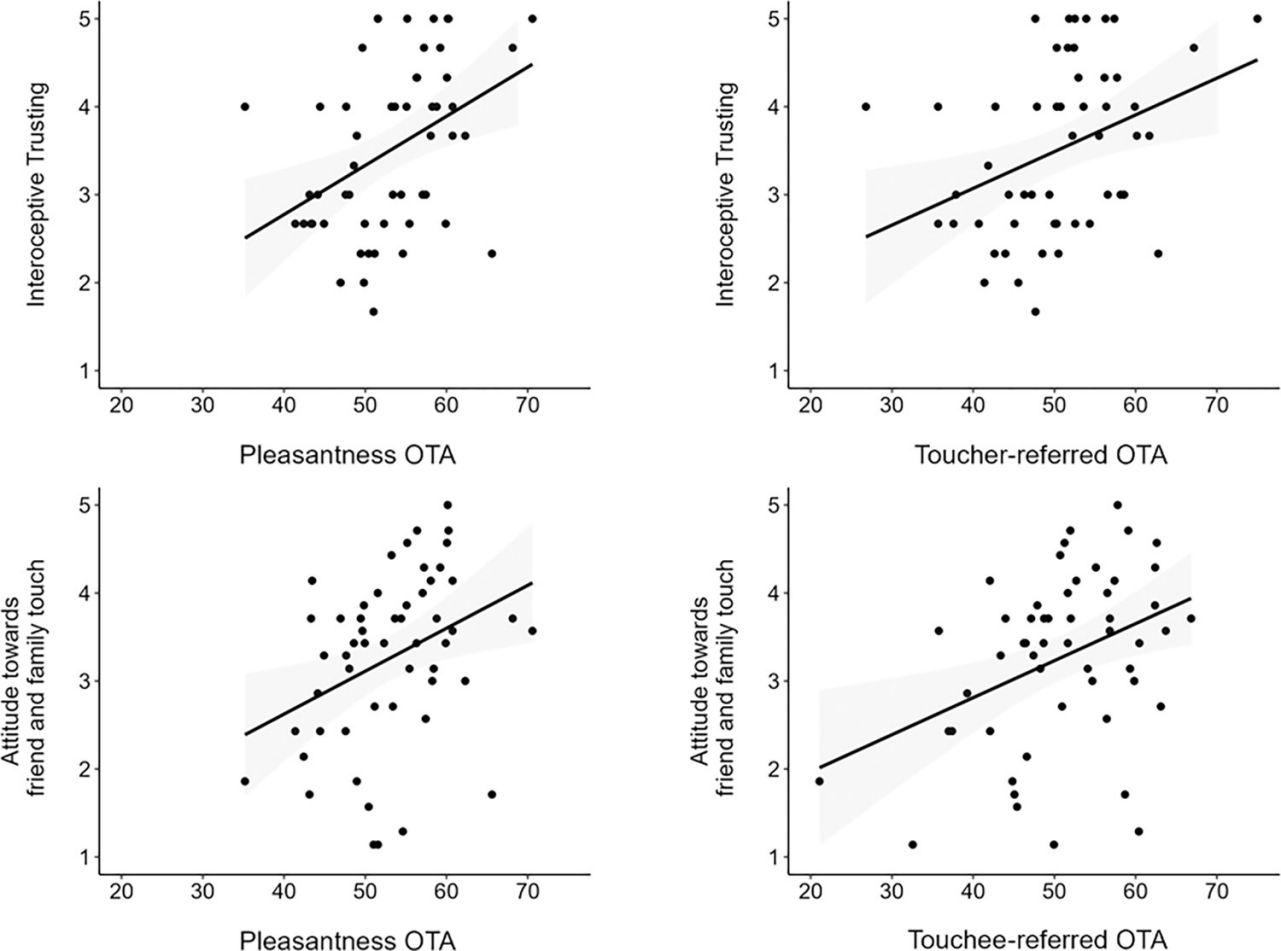
Scatter plots representing the significant correlations between OTA indexes and the questionnaire scales. Dots and represent observations; the shaded grey areas represent SE.

For the OTA indexes, higher scores obtained at the MAIA trusting scale were associated with higher appraisal for model-referred pleasantness-question (*r* = 0.41, *p* = 0.002) and toucher-referred (*r* = 0.36, *p* = 0.008) ratings. These indicated that the more participants trusted and felt as a safe place their own body, the higher they tended to appraise the touch pleasantness for another person, and for the person delivering the touch. In a similar vein, a more positive attitude towards friend and family touch correlated with higher ratings for model-referred pleasantness-question (*r* = 0.36, *p* = 0.009) and touchee-referred (*r* = 0.39, *p* = 0.004) ratings. Namely, the more positive participants’ attitude to receive interpersonal touch by family and friends, the more they judged positively the observed stroking for another person, and for the person receiving the touch. All other correlations were non-significant (all *r* < 0.34, all *p* > 0.017). The significant correlations are reported in Fig 3.

No significant correlations emerged between PTA and any of the questionnaire scales (all *r* < 0.25, all *p* > 0.083) (Table 2).

**Table 2:** Correlation analysis results. Spearman’s *r* and the corresponding *p*-value (in brackets) for each correlation between OTA and PTA indexes and the selected questionnaire scales. Asterisks indicate significant correlations after Bonferroni-correction (corrected *p* = 0.013).

	MAIA		TEAQ	
Index	Noticing	Trusting	Friend and family touch	Childhood touch
OTA Desirability	0.06 (0.663)	0.31 (0.024)	0.33 (0.018)	-0.001 (0.992)
OTA Pleasantness	0.24 (0.089)	0.41 (0.002)*	0.36 (0.009)*	0.01 (0.957)
OTA Toucher	0.08 (0.574)	0.36 (0.008)*	0.26 (0.058)	-0.06 (0.664)
OTA Receiver	0.18 (0.198)	0.32 (0.021)	0.39 (0.004)*	0.06 (0.658)
PTA Desirability	0.24 (0.092)	0.13 (0.372)	0.19 (0.167)	0.14 (0.307)
PTA Pleasantness	0.04 (0.757)	-0.03 (0.815)	0.10 (0.456)	0.24 (0.084)
PTA Toucher	0.13 (0.337)	-0.02 (0.880)	0.21 (0.137)	0.23 (0.096)
PTA Receiver	0.13 (0.343)	0.10 (0.469)	0.09 (0.510)	0.14 (0.324)

## Discussion

Previous research has found that reception of touch delivered at CT-optimal velocities is preferred over slower of faster stroking velocities, both when the participants directly receive the touch and when they see another person receiving it [20,22–25]. The present study aimed to compare the appraisal ratings of vicarious touch execution and reception. Participants preferred touch delivered at CT-optimal than non-optimal stroking velocities when they had to refer either to the toucher or the touchee of the observed tactile interaction. To the best of our knowledge, this is the first study documenting a CT-specific preference not only for vicarious reception [46], but also for vicarious delivering of interpersonal touch. These findings are in line with the hypothesis that humans are inherently ‘wired’ to receive and deliver affective touch [30,36], as well as to distinguish CT-optimal stroking when observing the reception and delivery of interpersonal touch [21], suggesting that vicarious perception of others both receiving and giving interpersonal touch is critical for adaptive behaviour in social contexts [39].

The result of a CT-specificity for vicarious delivery of touch suggests that observation of delivery of affective touch may activate simulative representations of the stroking gesture, which would help individuals with understanding the kind of tactile stimulation to be more appropriate to match the touchee’s needs [59]. Accordingly, previous neurophysiological research has found markers of simulation processes during vicarious touch perception [60–62] and when participants had to carry out a consoling touch on the partner [63]. However, it is less clear whether and how these simulative representations of the observed executed touch might map CT-specific features, such as the stroking velocity and the different CT-innervation of hairy and glabrous skin sites. Our result of a CT-specificity for vicarious delivery of touch might depend on the top-down expectations that affective touch would entail positive values also for the toucher [30], rather than reflecting an enhanced simulation for CT-optimal compared to non-CT optimal velocities. Thus, the neurocognitive mechanisms underpinning these behavioural results should be directly investigated in future studies, for instance testing whether CT-targeted touch modulates motor resonance processes [64].

Importantly, our findings pointed to a preference for vicarious touch reception compared to execution, which was specific for CT-optimal velocity. This preference reflects, at a vicarious representation level, a previous study documenting that being stroked was perceived as more pleasant than stroking at CT-optimal velocities [34]. Accordingly, such difference might depend on the scarce presence of CT fibres in the palm, so that vicarious reception would benefit more directly from CT-optimal stroking compared to vicarious execution. This view is also supported by present and previous results [23] that vicarious affective touch is perceived as more pleasant when received on or delivered to the hand-dorsum compared to the palm, according to a different distribution of CT fibres between hairy and glabrous skin [38,65]. That is, even though CT-specific preferences could be similarly elicited in glabrous and hairy skin sites [66], there is a functional difference between the skin typically involved in touch reception and the skin through which reaching out to touch [67,68]. This critical distinction, supported also by evidence of dissociable somatosensory responses to touch on hairy and glabrous skin [69], might result in the CT-specific advantage for touchee-related compared to toucher-related ratings reported here.

Furthermore, as the hedonic, positive value of a touch event would be determined by the matching between its perceived purpose and the goals of the touch receiver [70], vicarious reception of affective touch would be more important for the observer than vicarious execution in order to correctly infer affective and social information conveyed by interpersonal touch. Accordingly, an increasing number of studies documented a relationship between vicarious touch reception and affective processing, with a wide network of socio-cognitive and somatosensory areas contributing to understanding and, more importantly, feeling observed touch [19,20,39,71–75]. Of note, during observation of interpersonal touch events, activations in the insula and somatosensory cortices, which would support the “feel” of touch, are thought to capture the perceived social intentionality of stroking gestures [21,61,76,77], a mechanism that would help individuals to “resonate” with other’s affective experience of being touched [60,78,79].

Our correlation results partially support the hypothesis that diverse socio-affective mechanisms might underlie vicarious reception and execution of touch. The positive correlation between attitudes towards friend and family touch and touchee-referred ratings is in line with the rewarding value of social touch for the touchee [80]. Indeed, the more individuals are keen to engage in interpersonal tactile interactions with people close to them, the more touch reception gains a positive salience, and thus is rated as more pleasant [40,50]. In keeping with a previous study showing that interoceptive signals may affect touch delivery [81], higher toucher-referred ratings were also reported by individuals with higher trust in their own-body signals. Indeed, the way a person perceives the own bodily signals plays an active role in driving behaviour and predicting the consequences of one’s actions [82]. Hence, higher interoceptive awareness might help to understand which touch stimulation may match the touchee’s expectations, leading to increased appraisal judgments of interpersonal touch. It should be noted, however, that these correlations were not specific for CT-optimal velocity, since only the OTA (collapsing appraisal ratings across velocities), but not the PTA (reflecting selective preference for CT-optimal vs. non optimal velocities) index showed significant correlations. Thus, the associations might be related to general top-down mechanisms influencing vicarious touch perception rather than to the selective mapping of touch features related to the physiology of CT afferents [20,39].

Importantly, as expected from previous studies [23,24], a general advantage for model-referred pleasantness compared to self-referred desirability ratings of touch emerged for both toucher- and touchee-related judgments. This is in line with the idea that humans can rely on the learned value of social touch when judging touch pleasantness for a third person [20,39]. Notably, model-referred pleasantness ratings were significantly associated with both attitudes towards family and friend touch and interoceptive trusting. These results confirm that, even for vicarious delivery of touch, pleasantness judgements referred to a third person are influenced by top-down expectations on the hedonic value of interpersonal tactile stimulations [83].

Our study had limitations that should be carefully considered. First, a small part of the sample completed the procedures in the lab while the remaining participants completed the study online. Even though reliability of online data was supported by previous research using similar procedures [46], no assessment measures of concentration and engagement were included in the online testing, a limitation that must be acknowledged. However, the response time was consistent across subjects (mean = 2.692 ms, SD = 1.278 ms). Overall, it should be noted, the lab-based an online-based groups presented similar ratings across conditions, with only few conditions showing between-group differences higher than 10 rating points. These differences may depend on the expected high individual variability in touch appraisal [45], which may yield more extreme values in a small sample such as the lab-based one. However, the differences in the PTA and OTA indexes were all lower than, respectively, 0.10 and 10 rating points, thus reassuring on data similarity obtained from the two modalities. In a similar vein, this study did not consider many sociodemographic information, such as socioeconomic status, and was not homogeneous in terms of ethnic background as it recruited participants from two European countries (i.e., Italy and UK). For these reasons, caution is warranted in generalizing these findings across diverse populations. Although the use of different questions for self-referred desirability versus model-referred pleasantness was in accordance with previous studies [20,23,24], videos were not presented from a first-person viewing perspective. Future research should consider to add videos recorded from a first-person viewing perspective, so that participants would be shown the touch event as if they were the toucher or the touch receiver. Furthermore, the task was designed to avoid giving any clues about the context to focus on CT-related manipulations (i.e., velocity, body site). However, this way it did not consider contextual and social factors that may affect vicarious touch perception [70]. Finally, despite educated speculations about different neurocognitive mechanisms for vicarious touch reception and execution were advanced, this study exclusively adopted behavioural measures. Future research should investigate neurophysiological evidence of vicarious touch processing, as this topic has just started to be elucidated [39].

Limitations notwithstanding, this study is the first to provide evidence of a CT-specific preference for vicarious touch execution, and it hints at different cognitive and affective mechanisms which might subserve simulation of vicarious reception and execution of social touch.

## Supporting information

S1 File On-line and lab-based participants’ performance. S1 and S2 Tables reporting mean (SD) for each condition and for the OTA and PTA indexes in the on-line recruitment and lab-based recruitment groups and between-group delta.
(DOCX)
