# Supplementary material for: To touch or to be touched? comparing appraisal of vicarious execution and reception of interpersonal touch
Source: PLoS One. 2024 May 17;19(5):e0293164. doi: 10.1371/journal.pone.0293164 (PMC11101113; doi:10.1371/journal.pone.0293164)
Supplement: S1 File — S1 and S2 Tables reporting mean (SD) for each condition and for the OTA and PTA indexes in the on-line recruitment and lab-based recruitment groups and between-group delta. (DOCX) [file pone.0293164.s001.docx]

**Supplementary table 1**. Mean (SD) for each condition in the on-line recruitment and lab-based recruitment groups and between-group delta.

|  |  |  |  | **Recruitment** | |  |
| --- | --- | --- | --- | --- | --- | --- |
| **Agent** | **Question** | **Velocity** | **Skin site** | **Lab-based** | **Online** | **Delta** |
| Toucher | Desirability | Static | Hand | 53 (17) | 48 (16) | -5 |
|  |  |  | Palm | 57 (22) | 49 (15) | -8 |
|  |  | CT-optimal | Hand | 69 (15) | 62 (16) | -7 |
|  |  |  | Palm | 78 (16) | 57 (18) | -20 |
|  |  | Fast | Hand | 30 (24) | 32 (17) | 1 |
|  |  |  | Palm | 37 (26) | 32 (16) | -5 |
|  | Pleasantness | Static | Hand | 52 (11) | 52 (14) | 0 |
|  |  |  | Palm | 56 (11) | 53 (12) | -4 |
|  |  | CT-optimal | Hand | 71 (9) | 67 (13) | -4 |
|  |  |  | Palm | 70 (13) | 35 (16) | -5 |
|  |  | Fast | Hand | 46 (17) | 41 (14) | -4 |
|  |  |  | Palm | 46 (22) | 38 (15) | -7 |
| Receiver | Desirability | Static | Hand | 49 (20) | 49 (15) | 0 |
|  |  |  | Palm | 56 (14) | 49 (15) | -8 |
|  |  | CT-optimal | Hand | 77 (8) | 66 (16) | -10 |
|  |  |  | Palm | 75 (10) | 63 (20) | -12 |
|  |  | Fast | Hand | 38 (24) | 30 (16) | -8 |
|  |  |  | Palm | 40 (25) | 29 (19) | -11 |
|  | Pleasantness | Static | Hand | 53 (18) | 50 (16) | -2 |
|  |  |  | Palm | 60 (9) | 51 (14) | -9 |
|  |  | CT-optimal | Hand | 75 (7) | 69 (13) | -6 |
|  |  |  | Palm | 77 (6) | 66 (17) | -11 |
|  |  | Fast | Hand | 45 (12) | 38 (15) | -7 |
|  |  |  | Palm | 51 (21) | 36 (16) | -14 |

**Supplementary table 2**. Mean (SD) for the OTA and PTA indexes in the on-line recruitment and lab-based recruitment groups and between-group delta.

|  | **Recruitment** | |  |
| --- | --- | --- | --- |
| **Index** | **Lab-based** | **Online** | **Delta** |
| OTA Desirability | 55 (8) | 47 (9) | 8 |
| OTA Pleasantness | 58 (6) | 52 (7) | 6 |
| OTA Toucher | 55 (7) | 50 (8) | 5 |
| OTA Receiver | 58 (6) | 50 (9) | 8 |
| PTA Desirability | 0.78 (0.73) | 0.69 (0.48) | 0.09 |
| PTA Pleasantness | 0.48 (0.39) | 0.55 (0.39) | 0.07 |
| PTA Toucher | 0.65 (0.69) | 0.55 (0.41) | 0.1 |
| PTA Receiver | 0.60 (0.45) | 0.69 (0.45) | 0.09 |
